# Bone marrow-derived and peritoneal macrophages have different inflammatory response to oxLDL and M1/M2 marker expression – implications for atherosclerosis research

**DOI:** 10.1038/srep35234

**Published:** 2016-10-13

**Authors:** Line S. Bisgaard, Christina K. Mogensen, Alexander Rosendahl, Helena Cucak, Lars Bo Nielsen, Salka E. Rasmussen, Tanja X. Pedersen

**Affiliations:** 1 Dept. of Biomedical Sciences, University of Copenhagen, Denmark; 2 Dept. of Diabetic Complication Biology, Novo Nordisk, Denmark; 3 Dept. of Biopharmaceuticals New Heamophilia, Novo Nordisk, Denmark; 4 Dept. of Clinical Biochemistry, Copenhagen University Hospital Rigshospitalet, Denmark; 5 Dept. of ADME, Novo Nordisk, Denmark

## Abstract

Macrophages are heterogeneous and can polarize into specific subsets, *e.g.* pro-inflammatory M1-like and re-modelling M2-like macrophages. To determine if peritoneal macrophages (PEMs) or bone marrow derived macrophages (BMDMs) resembled aortic macrophages from ApoE−/− mice, their M1/M2 phenotype, inflammatory status, and lipid metabolism signatures were compared. oxLDL accumulation was similar in PEMs and BMDMs. On protein expression level, BMDMs showed an M2-like CD206^high^CD11c^low^ profile, while cholesterol loading led to enhanced CD11c expression and reduced MCP-1 secretion. In contrast, PEMs expressed low levels of CD206 and CD11c, and responded to cholesterol loading by increasing CD11c expression and MCP-1 secretion. mRNA expression of M1/M2 markers was higher in PEMS than BMDMs, while lipid metabolism genes were similarly expressed. Whole aorta flow cytometry showed an accumulation of M2-like CD206^high^CD11c^low^ macrophages in advanced versus early atherosclerotic disease in ApoE−/− mice. In isolated lesions, mRNA levels of the M2 markers Socs2, CD206, Retnla, and IL4 were downregulated with increasing disease severity. Likewise, mRNA expression of lipid metabolism genes (SREBP2, ACSL1, SRB1, DGAT1, and cpt1a) was decreased in advanced versus early lesions. In conclusion, PEMs and BMDMs are phenotypically distinct and differ from macrophages in lesions with respect to expression of M1/M2 markers and lipid metabolism genes.

Macrophages play a key role in atherogenesis, and the overall macrophage functionality is critical for the balance between plaque progression and regression. Thus, macrophages take up oxidized low density lipoproteins (oxLDL), form foam cells, and secrete inflammatory mediators, and thereby induce plaque progression. On the other hand, macrophages can also efflux cholesterol to high density lipoproteins, enabling reverse cholesterol transport and potentially plaque regression^1^,^2^.

Macrophages are heterogeneous and express one or more pan-macrophage markers such as Ly6c, F4/80, and CD68 based on tissue of origin, maturity, and activation status^3^,^4^,^5^,^6^. Furthermore, macrophages undergo polarization towards different phenotypes dependent on their local microenvironment. *In vitro*, macrophages are classified as classically activated (M1) or alternatively activated (M2) macrophages. M1 macrophages have pro-inflammatory effects, i.e. secretion of pro-inflammatory cytokines (e.g. tumor necrosis factor (TNF)α and monocyte chemoattractant protein (MCP)-1) and activation of endothelial cells leading to attraction of monocytes^7^,^8^. M2 macrophages are involved in tissue remodelling and fibrosis^7^. While the expression patterns of inflammatory genes in M1 *vs* M2 macrophages are relatively well described, the consensus regarding the M1/M2 specific expression patterns of genes involved in lipid metabolism is less clear^8^. Functionally, it has been shown that M2 macrophages take up oxLDL to a higher degree than M1 macrophages^9^ although contradictory results exist^8^. Nevertheless, the *in vitro* functionalities of M1 and M2 macrophages suggest that the M1/M2 macrophage profile affects atherogenicity.

Different markers are used to identify M1 (e.g. TNFα, iNOS, IL6 and CD11c) and M2 (e.g. CD206, Arg1 and Retnla) macrophages, respectively^7^,^8^, and new markers are continuously being detected^10^. In human atherosclerotic plaques markers for both M1 and M2 macrophages are present both in the early and advanced stages^11^. M1 macrophages dominate in the shoulder regions, whereas M2 macrophages are mostly found in the adventitia^11^. Cells expressing M1 and/or M2 markers are also present in murine atherosclerotic plaques^12^,^13^ and it has been suggested that there is a phenotypic shift from M2 to M1 with plaque progression^12^,^13^. Presently, it is not fully elucidated whether M2 macrophages attenuate or augment atherogenesis. Thus, studies in hypercholesterolemic mice suggest that a shift towards M2 macrophages reduces atherosclerosis^14^,^15^,^16^ whereas knock out of the interleukin (IL)4 gene, which is known to induce M2 polarisation, reduces plaque progression^17^. Combined, this indicates that the balance between M1 and M2 macrophages is indeed important for plaque progression, at least in mice.

The majority of studies on M1/M2 macrophage polarization have been conducted *in vitro*. Two of the most commonly used *in vitro* macrophage models in atherosclerosis research include bone marrow derived (BMDM) and peritoneal (PEM) macrophages. Despite the importance of the phenotype for macrophage functionality, the M1/M2 profile/phenotype is often not addressed when either BMDMs or PEMs are used to analyse atherogenic properties.

The purpose of this study was to address the phenotype of BMDMs and PEMs and compare the M1/M2 profiles to that of macrophages in whole aortas and/or isolated atherosclerotic lesions of ApoE−/− mice.

## Results

### *In vitro* foam cell formation and inflammatory response to oxLDL stimulation

**Figure 1:**
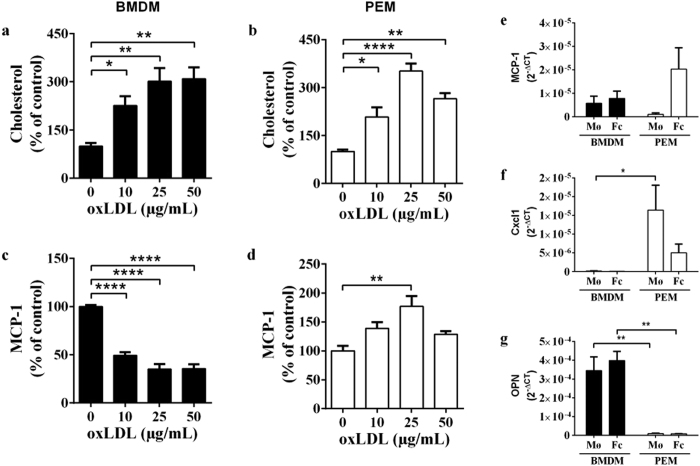
BMDMs and PEMs have a different inflammatory response to oxLDL stimulation. (**a–d**) Cholesterol or MCP-1 content (% of control (0 μg/mL oxLDL)) in BMDMs (**a,c**) and PEMs (**b,d**) after stimulation with 0–50 μg/mL oxLDL for 24 hours (each graph represents 3 separate experiments (n = 3–4 wells per experiment)). *p < 0.05, **p < 0.01, ****p < 0.0001, as determined by 1-way ANOVA with Dunnets post-test. Each graph represents one representative experiment (triplicate analyses of BMDMs from one mouse or PEMs from a pool 4–5 of mice). Experiments were triplicated with similar results (i.e. n = 3 mice for BMDMs and 3 pools of 4–5 mice for PEMs). (**e–g**) mRNA expression (2^−ΔCT^) of monocyte chemoattractant protein 1 (MCP-1) (**e**), chemokine (C-X-C motif) ligand 1 (Cxcl 1) (**f**) or osteopontin (OPN) (**g**) in BMDMs or PEMs after incubation with 0 μg/mL oxLDL (macrophage, Mø) or 25 μg/mL oxLDL (foam cells, Fc) for 24 hours (n = 3 mice for BMDMs; n = 3 pools of mice for PEMs). *p < 0.5, **p < 0.01, as determined by 2-way ANOVA.

To assess the functionality of BMDMs versus PEMs, we isolated bone marrow and peritoneal exudate from mice. Bone marrow derived cells were differentiated to macrophages, whereas PEMs were used freshly. Accumulation of cholesterol upon oxLDL stimulation peaked at 25 μg/mL oxLDL, and was similar in PEMs and BMDMs (Fig. 1a,b).

BMDMs reduced their MCP-1 secretion by ~65% (p < 0.0001; 0 vs 25 μg/mL) upon oxLDL stimulation, while the release from PEMs was increased by ~77% (p < 0.0015; 0 vs 25 μg/mL) (Fig. 1c,d). A trend towards increased MCP-1 mRNA expression was observed in PEMs upon foam cell formation (25 μg/mL oxLDL) (Fig. 1e). Chemokine (C-X-C motif) ligand 1 (cxcl1, aka KC) was expressed almost exclusively in PEMs and expression was reduced upon foam cell formation (Fig. 1f), whereas osteopontin (OPN) was mainly expressed in BMDMs (Fig. 1g).

Thus, despite similar cholesterol accumulation, the inflammatory response to oxLDL exposure was remarkably different in PEMs and BMDMs.

### M1/M2 marker expression in BMDMs and PEMs

To address whether the different inflammatory profiles in PEMs and BMDMs were related to their M1/M2 polarization signature, we analysed expression of the M1 marker CD11c and the M2 marker CD206 by flow cytometry on CD68^+^F4/80^+^ and CD68^−^F4/80^+^ macrophages (gating strategy in Suppl. Fig. 6).

**Figure 2:**
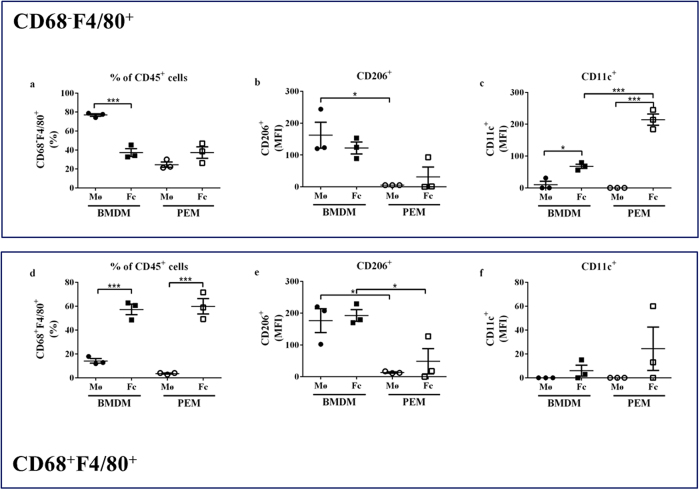
M1/M2 marker expression in BMDMs and PEMs. (**a–f**) Flow cytometry of BMDMs and PEMs after incubation with 0 μg/mL oxLDL (macrophage, Mø) or 25 μg/mL oxLDL (foam cells, Fc) for 24 hours (n = 3 mice for BMDMs and 3 pools of 4–5 mice for PEMs). For both single positive (CD68^−^F4/80^+^) and double positive (CD68^+^F4/80^+^) cells, the percentage of CD45^+^ cells (**a,d**), and MFI for CD206 (**b,e**) and CD11c (**c,f**) expression were detected. MFI: Mean Fluorescent Intensity. *p < 0.05, ***p < 0.001 as determined by 1-way ANOVA with Tukey’s post-test.

The majority of BMDMs (80%) were CD68^−^F4/80^+^, whereas 15% were CD68^+^F4/80^+^ (Fig. 2a,d). In contrast, only 20% of PEMs were CD68^−^F4/80^+^, and almost none were CD68^+^F4/80^+^ (Fig. 2a,d). Upon oxLDL stimulation, the fraction of CD68^−^F4/80^+^ BMDMs was reduced 2-fold, concomitant with a 3-fold increase of CD68^+^F4/80^+^ cells (Fig. 2a,d). In parallel, the majority of the PEMs acquired a CD68^+^F4/80^+^ profile upon oxLDL exposure (Fig. 2d).

CD206 expression was significantly higher in BMDMs compared to PEMs in both CD68^−^F4/80^+^ and CD68^+^F4/80^+^ macrophages (Fig. 2b,e). This was not modulated by oxLDL stimulation (Fig. 2b,e). Basal expression of CD11c protein was low in BMDMs and barely detectable in PEMs in both CD68^−^F4/80^+^ and CD68^+^F4/80^+^ macrophages (Fig. 2c,f). Foam cell formation significantly enhanced expression of CD11c in CD68^−^F4/80^+^ BMDMs, and this effect was even more pronounced in CD68^−^F4/80^+^ PEMs (Fig. 2c). Interestingly, CD11c was neither expressed by CD68^+^F4/80^+^ PEMs nor by CD68^+^F4/80^+^ BMDMs (Fig. 2f), and foam cell formation did not change this (Fig. 2f). Taken together, this pointed towards a different polarization of PEMs and BMDMs.

### M1/M2 marker gene expression and the effect(s) of foam cell formation

To further explore the polarization signature of PEMs and BMDMs, we constructed a qPCR array, and analysed M1/M2 mRNA expression patterns as well as expression of lipid metabolism genes (see Table 1 for a list of genes).

Similarly to the flow cytometry result (Fig. 2), we found that CD206 was expressed to a lower extent in PEMs as compared to BMDMs (Suppl. Fig. 1 and Table 1, columns 2 and 3). For CD11c, there was a trend towards higher expression in PEMs as compared to BMDMs. Foam cell formation did not affect mRNA levels of either CD206 or CD11c (Suppl. Fig. 1 and Table 1, columns 4 and 5).

**Figure 3:**
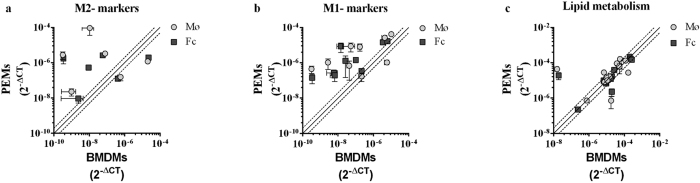
No clear M1/M2 gene expression profile in BMDMs and PEMs. (**a–c**) mRNA levels (2^−ΔCT^) of M2 markers (**a**), M1 markers (**b**), or genes related to lipid metabolism (**c**) in PEMs depicted as a function of mRNA levels (2^−ΔCT^) in BMDMs (n = 3 mice for BMDMs and 3 pools of 4–5 mice for PEMs). Each grey circle represents expression of a given gene in macrophages (Mø, 0 μg/mL oxLDL 24 h), whereas each square represents expression of a given gene in foam cells (Fc, 25 μg/mL oxLDL 24 h) as measured by PCR array analyses. The full line indicate genes, with similar expression levels in both cell types, while data points placed above or below the dotted lines are more than 2 fold up- or down-regulated, respectively, in PEMs as compared to BMDMS. The specific genes of interest, their fold change, and statistics can be found in Table 1.

To address whether this pattern was maintained if all M1 and M2 marker genes were taken into account, the gene expression levels in PEMs were depicted as a function of the gene expression levels in BMDMs (Fig. 3). In this plot, the full line indicates genes, where the expression level is the same in the 2 cell types, while data points placed above or below the dotted lines are more than 2 fold up- or downregulated, respectively, in PEMs as compared to BMDMS. The specific genes of interest, their magnitude of expression, and fold change can be found in Suppl. Fig. 1 and Table 1. When all markers were taken into account, neither PEMs nor BMDMs had a clear M2 or M1 phenotype profile (Fig. 3a,b). Instead, PEMs in general had a much higher expression level of both M2 and M1 markers compared to BMDMs both for macrophages and foam cells (Fig. 3a,b). In contrast, the expression pattern of lipid metabolism genes was strikingly similar in PEMs and BMDMs (Fig. 3c). The only exceptions were lectin-type oxidized LDL receptor 1 (LOX1), which was expressed higher in PEMs compared to BMDMs (Table 1, columns 2 and 3, and Suppl. Fig. 1) and peroxisome proliferator-activated receptor (PPAR)ɣ which was expressed higher in BMDMs compared to PEMs (Table 1, columns 2 and 3, and Suppl. Fig. 1).

Next, the effect of foam cell formation on gene expression in PEMs and BMDMs was addressed. The M1 associated marker IP10 (Cxcl10) was significantly decreased upon foam cell formation in both PEMs and BMDMs, whereas the M1 associated marker TNFα was decreased only in PEMs (Suppl. Fig. 1 and Table 1, columns 4 and 5). In contrast, several lipid metabolism genes were affected by foam cell formation. In PEMs, foam cell formation led to a significant reduction of mRNA encoding macrophage scavenger receptor 1 (MSR1), Scavenger receptor class B member 1 (SRB1), and Sterol Regulatory Element-Binding Protein 2 (SREBP2), while ATP-binding cassette sub-family G member 1 (Abcg1) expression was upregulated (Table 1, column 4, and Suppl. Fig. 1). Notably, none of these genes were affected by foam cell formation in BMDMs. Instead, we found a significant upregulation of Sterol Regulatory Element-Binding Protein 1 (SREBP1) and a reduction of diglyceride acyltransferase (DGAT2) expression in BMDMs upon foam cell formation (Table 1, column 5 and Suppl. Fig. 1).

Overall, the gene expression patterns indicated that neither PEM nor BMDM macrophages had a clear M1 or M2 profile. Furthermore, foam cell formation did not result in M1 or M2 polarisation of either PEMs or BMDMs. Foam cell formation did, however, affect expression of genes involved in lipid metabolism, particularly in PEMs.

### Macrophage M1/M2 profile in whole aortas from ApoE−/− mice

To relate the *in vitro* findings to macrophage phenotype *in vivo*, we performed flow cytometry analyses of whole aortas from ApoE−/− mice 10 and 17 weeks after switch to a western type diet (WD). Plasma cholesterol levels were identical after 10 and 17 weeks on diet, whereas plasma triglycerides were significantly lower in 17 vs. 10 weeks of diet (Suppl. Table 1).

**Figure 4:**
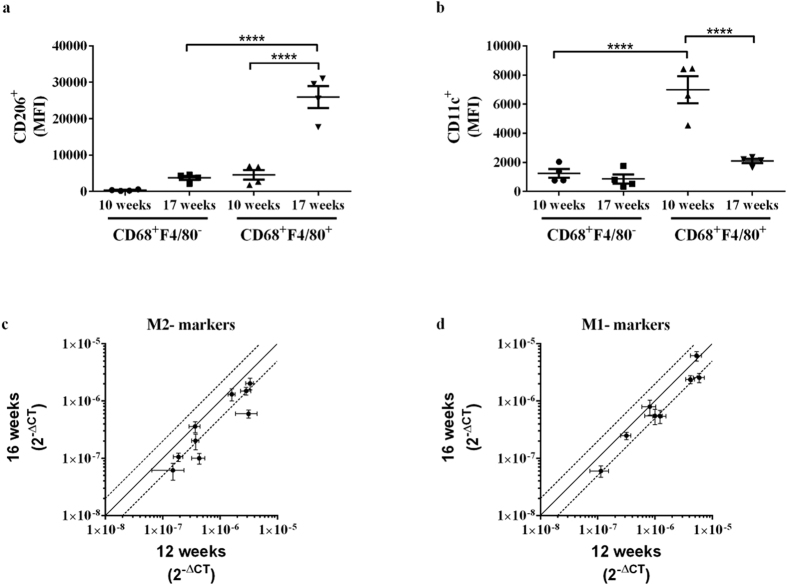
Macrophage polarisation in whole aortas and isolated lesion areas from ApoE−/− mice. (**a,b**) Flow cytometry of whole aortas to detect CD206 (**a**) and CD11c (**b**) expression (MFI: mean fluorescent intensity) in CD68^+^F4/80^−^ and CD68^+^F4/80^+^ cells after 10 or 17 weeks on WD (n = 4 mice/time point). *p < 0.05, ****p < 0.0001 as determined by 1-way ANOVA with Tukey’s post-test. See Suppl. Fig. 3 for representative MFI histograms. (**c,d**) mRNA expression levels (2^−ΔCT^) of M2 (**c**) or M1 markers (**d**) in aortic lesion areas isolated after 12 or 16 weeks of WD (n = 7 mice/group). Each point represents expression level (2^−ΔCT^) of a given gene. The full line indicates genes, where the expression level is the same, while data points placed above or below the dotted lines are more than 2 fold up- or down-regulated, respectively, after 16 compared to 12 weeks of WD. The specific genes of interest, their fold change and statistics can be found in Suppl. Table 2.

As expected, there was an overall increase in the frequency of macrophages within the leukocyte population in whole aortas after 17 as compared to 10 weeks on WD (Suppl. Fig. 2). The macrophages were divided into 2 distinct sub-populations; F4/80^−^CD68^+^ or F4/80^+^CD68^+^. The frequency of F4/80^+^CD68^+^ macrophages was significantly increased with time, while the frequency of F4/80^−^CD68^+^ macrophages was non-significantly upregulated (Suppl. Fig. 2). Interestingly, expression of CD206 (M2) was increased, in the F4/80^+^CD68^+^ macrophages, whereas expression of CD11c (M1) was decreased after 17 weeks compared to 10 weeks of WD (Fig. 4a,b). In the F4/80^−^CD68^+^ macrophages, no change in CD206 or CD11c expression was detected over time (Fig. 4a,b) (gating strategy in Suppl. Fig. 3). Combined, the flow cytometry data indicate, that there is a skewing towards a more M2-like profile with disease progression in whole aortas.

### Gene expression in isolated atherosclerotic lesions from ApoE−/− mice

In order to analyse the specific content of M1/M2-like macrophages in atherosclerotic lesions, we isolated lesion and non-lesion areas from ApoE−/− mice after 12 and 16 weeks on a WD diet. Expression of the pan macrophage marker galectin-3 was significantly higher in lesion vs non-lesion areas, thus suggesting that the isolated lesion areas were indeed macrophage-rich atherosclerotic lesions (Suppl. Fig. 4). Next, we used qPCR arrays to analyse gene expression of M1/M2 markers and lipid metabolism genes in the lesion areas (Suppl. Table 2 and Suppl. Fig. 5). Plasma cholesterol levels were increased, whereas plasma triglycerides were similar, after 16 vs 12 weeks on diet (Suppl. Table 1).

To compare gene expression early and late, we plotted gene expression of M2 and M1 markers in 16 week samples against gene expression in 12 week samples (Fig. 4c,d). Both M2 and M1 markers were expressed in the lesions early and late (Fig. 4c,d, Suppl. Table 2, and Suppl. Fig. 5). Interestingly, we found a small, but significant, overall reduction of M2 genes over time (p = 0.03, Hotelling’s T^2 test). For lipid metabolism genes, we observed down-regulation of SRB1, SREBP2, diglyceride acyltransferase 1 (DGAT1), Long-chain-fatty-acid—CoA ligase 1 (ACSL1) and carnitine palmitoyltransferase 1a (cpt1a) after 16 weeks compared to 12 weeks on diet (Suppl. Table 2 and Suppl. Fig. 5).

Thus, both M1 and M2 genes are expressed in atherosclerotic lesions both early and late, and an overall trend towards downregulation of M2-associated genes was observed with disease progression.

## Discussion

Atherosclerosis is a progressive silent disease which builds up during decades before presenting with clinical symptoms^18^. During this asymptomatic phase, the local composition of leukocytes and particularly macrophages probably changes significantly. Macrophages are described to be highly pleiotropic with a capacity to rapidly shift polarization profile due to subtle changes in their microenvironment^8^,^19^. Since the macrophage phenotype(s) present in atherosclerotic lesions are believed to affect atherogenesis, it is relevant to explore which macrophages are present in lesions *in vivo*. Moreover, it is important to understand the *in vitro* models that are used to mimic plaque macrophages in greater detail.

We have addressed the phenotype composition and functionality of 2 widely used *in vitro* macrophage models in atherosclerosis research, i.e. BMDMs and PEMs, and compared them to aorta derived macrophages from ApoE−/− mice with initial and established disease to elucidate whether any of the two constitute a more appropriate model for plaque associated macrophages.

On protein expression level, BMDMs presented with an M2-like phenotype (CD206^high^CD11c^low^). In contrast, PEMs were only weakly CD206 positive and CD11c negative. This relatively simplistic view of an M2-like profile in BMDMs was corroborated by gene expression of LOX-1 and PPARγ. Thus, LOX-1 is known to be expressed at higher levels in M1^9^ and PPARγ^20^ at higher levels in M2 macrophages, thus matching the findings in BMDMs in our study. These findings might reflect the use of M-CSF for BMDM maturation, as others have shown that M-CSF stimulation induces an M2-like phenotype in macrophages^21^.

Transcriptional evaluation confirmed higher expression of both CD206 and of PPARγ (which has been shown to be important for M2 differentiation^22^) on BMDMs compared to PEMs, but the remaining M2 markers detected in BMDMs, i.e. Arg1, Chis3l1, Socs2, and Ccl17, were not expressed at higher levels in BMDMs compared to PEMs. Instead, PEMs showed overall higher mRNA levels of both M1-like and M2-like genes compared to BMDMs. To our knowledge, the M1/M2 mRNA expression profile of BMDMs and PEMs has not been directly compared previously. However, a few functional studies have directly compared BMDMs and PEMs, and shown that they have different phagocytosis abilities^23^, different glucosaminoglycan secretion pattern^24^ and different migration abilities^25^. Combined, our study and the previously published studies highlight the fact that BMDMs and PEMs are inherently different both in terms of M1/M2 polarization signatures and functionality. These differences may be partly explained by the fact that one system uses freshly isolated cells (PEMs), and the other uses cells differentiated in culture (BMDMs).

As expected, we found that the frequency of macrophages in whole aortas increased during disease progression in ApoE−/− mice. The accumulated F4/80^+^CD68^+^ macrophages in advanced disease had significantly increased protein expression of CD206 (M2) and reduced expression of CD11c (M1). In contrast, when analysing mRNA levels in isolated atherosclerotic lesions, an overall reduction of both M1 and M2 markers, which reached significance for 4 M2 markers, was demonstrated in advanced vs early disease. The findings in whole aorta vs lesion areas do not necessarily contradict, since one is a measure of protein expression and the other is at RNA level. More importantly, however, the cell type composition in whole aortas differs significantly from the cell type composition in isolated lesion areas. Thus, in whole aortas, the abundance of macrophages is much smaller than in an atherosclerotic lesion. Moreover, it has been found that M1 macrophages localise mainly in the shoulder regions of plaques, while M2 macrophages are predominantly found in the adventitia^11^ and in non-lesioned areas of aorta^26^, at least in humans, thus fitting nicely with the results we have obtained. In isolated lesions, one might have expected a skewing towards a pro-inflammatory, ‘M1-like’, gene expression profile. Thus, Kadl *et al.* find that M1 macrophages predominate in plaques^13^, while Khalou-Lashet *et al.* show that M2 macrophages predominate in early lesions, while M1 macrophages are the most abundant in late lesion^12^. It is, however, difficult to compare their studies directly to ours, since there are important differences in the mouse strain used, the mouse diet, the time point analysed, the methodology used to distinguish the specific M1/M2 phenotype, and the number of markers used to ascertain a given M1/M2 phenotype. An additional layer of complexity arises from the fact that both M1 and M2 macrophages are likely found in the same atherosclerotic lesions. This was supported by our study, since we found expression of a number of both M1 and M2 markers in lesion areas, thus mimicking results obtained by other groups^11^,^12^,^13^. It should be noted, that our mRNA analyses cannot distinguish whether M1 and M2 markers are co-expressed by the same macrophages or whether distinct M1 and M2 macrophages co-exist within the plaque area. Finally, monocyte and macrophage origin is important for polarisation and functionality of macrophages during inflammation. It is possible that the phenotypic differences in aortic macrophages observed in the present study might relate to differences in origin^27^.

Cholesterol loading and subsequent foam cell formation is a hallmark of atherosclerosis. We addressed the effects of foam cell formation on mRNA expression of lipid metabolism genes in PEMs and BMDMs *in vitro* and analysed the expression of the same genes in isolated lesions from early and advanced atherosclerotic disease. *In vivo*, mRNA expression of ACSL1, SREBP2, SRB1, DGAT, and cpt1a was downregulated in isolated lesions. If translated to protein levels, these changes would functionally favour both prevention of further lipid accumulation (decreased biosynthesis of cholesterol (SREBP2), reduced triglyceride synthesis (DGAT)), and increased cholesterol accumulation (decrease in cholesterol efflux (SR-B1), and FFA oxidation (ACSL1 and cpt1a)). These findings highlight the cellular heterogeneity and complexity of atherosclerotic lesions.

*In vitro*, we found that cholesterol loading was similar in PEMs and BMDMs, whereas the effect of oxLDL on mRNA expression of lipid metabolism gene was markedly different. Thus, in PEMs, SREBP2, MSR1, and SR-B1 were downregulated upon foam cell formation, whereas Abcg1 expression was upregulated. In BMDMs, DGAT2 mRNA levels were significantly decreased, while SREBP1 levels were significantly upregulated. A previous study investigating the effects of cholesterol loading on expression of lipid metabolism genes in human monocyte derived macrophages, also detected decreased SREBP2 expression upon lipid loading^28^, thus mimicking the results obtained in PEMs in our study. This study also analysed mRNA expression of selected lipid metabolism genes *in vivo* (in human atherosclerotic lesions vs normal artery) and concluded, amongst others, that there was only partial similarity between changes in expression levels of lipid metabolism genes during plaque progression *in vivo* and lipid loading *in vitro*^28^. Although the 2 studies are difficult to compare directly, since there are differences in both the study setup, and the genes analysed, the fact that both studies only detect partial similarity of findings *in vivo* and *in vitro* highlights the difficulty in comparing, and trying to decipher, *in vivo* pathological pathways using *in vitro* model systems.

Recent studies point towards a link between lipid metabolism and macrophage polarization^29^. Thus, stimulation of M2 polarized macrophages with oxLDL leads to a skewing towards M1 macrophages^9^, while incubation with minimally oxidized LDL results in expression of pro-inflammatory cytokines in PEMs^18^. Moreover, ACSL deficient mice have been shown to express significantly reduced levels of M1-associated cytokines during diabetic conditions^30^, while PPARɣ expression is necessary for M2 differentiation^22^.

oxLDL stimulation increased CD11c expression, particularly in PEMs, thus indicating M1-skewing. Apart from this, cholesterol loading only led to minor/subtle differences in polarization signatures in PEMs and BMDMs. Thus, our observations indicate that oxLDL does not induce skewing towards either M1 or M2 macrophage phenotypes – regardless of the initial polarization signature of the cell type investigated. This is in line with both clinical observations from human atherosclerotic subjects, where plaques enriched in foam cells co-express M1- and M2-associated markers^11^, and mouse studies^31^.

## Conclusion

In conclusion, we have measured both functionality (i.e. lipid uptake and cytokine secretion) of PEMs and BMDMs, and characterised their protein marker and gene expression profile with the overall purpose of comparing their phenotype to the macrophage phenotype found in atherosclerotic lesions. If we only address one factor, e.g. the protein marker expression, our results indicate that BMDMs reflect the *in vivo* macrophage, at least at the later stages of lesion development, where the number of M2 macrophages detected by flow cytometry is increased. Importantly, however, our gene expression analyses clearly show that cells within atherosclerotic lesions express a multitude of both M1 and M2 markers. It would therefore be far too simplistic to base an M1/M2 characterisation on a single, or even a few, markers alone. If we take all of our data into account, it is not possible to determine whether BMDMs or PEMs constitute the best/most accurate *in vitro* model to study plaque associated macrophages, since (*i*) atherosclerotic lesions probably contain a heterogeneous macrophage population and (*ii*) neither PEMs nor BMDMs accurately matches the heterogeneity observed in *vivo* at least in our study. Based on our observations, caution should be taken when translating *in vitro* obtained macrophage results into a disease context. The inherent heterogeneity of macrophages and their intrinsic capacity to re-polarize rapidly based on subtle micro-environmental changes might prohibit the perfect model to be generated. However, detailed analysis of the local tissue derived cell types such as performed in our study will provide instrumental value to study specific functional mechanisms although the full disease context might not fully translate.

## Materials and Methods

### Cell culture

*BMDMs* were isolated from female C57BL/6 mice by flushing the femur and tibia with PBS. The bone marrow cells were resuspended in RPMI-1640 (Gibco, 31870) containing 1% L-glutamine, 1% Pen/Strep, MEM, NEAA x 1, 1% pyruvate and 20% FBS and 100 ng/mL M-CSF. Cells were incubated for 10 days at 37 °C and 5% CO_2_ with medium change every 3–4 day. For experimental setups, BMDMs were plated with a density of 11.500 cells/cm^2^ and stimulated with oxLDL (Intracel, conc. indicated in figure legends) for 24 hours in FBS and M-CSF free RPMI medium containing 0.2% BSA.

PEMs were isolated from the peritoneal cavity of female C57BL/6 mice by flushing the peritoneal cavity with 5 mL ice cold PBS. The retracted fluid was placed on ice until further processing. Cells were centrifuged and resuspended in serum free RPMI-1640 (Gibco, 12633) with 1% L-glutamine and 1% Pen/Strep, pooled and plated with a density of 23000 cells/cm^2^ and incubated at 37 °C and 5% CO_2_ 1–2 hours for PEMs to adhere. Subsequently, non-adherent cells were washed away with PBS and PEMs were incubated in RPMI with 10% FBS over night before stimulation. Samples with contamination of red blood cells were discharged. For cholesterol and MCP-1 protein measurements, cells were stimulated with oxLDL for 24 h in medium with 10% serum. For other experiments, the medium was changed to serum free RPMI containing 0.2% BSA cells at stimulation with oxLDL (conc. indicated in figure legends).

### MCP-1 and cholesterol measurements in cultured cells

For MCP-1 measurements, conditioned medium was collected from PEMs and BMDMs, centrifuged and transferred to new plates to avoid contamination from dead cells and frozen at −80 °C. MCP-1 was measured in the conditioned medium by alphaLISA analysis (Perkin Elmer).

Cholesterol was extracted with hexan:isopropanol (3:2), evaporated overnight in a fume hood and resuspended in isopropanol/1% Triton X-100. The cholesterol concentration was determined enzymatically with CHOD-PAP reagent (Roche/Hitachi), while proteins were extracted with 0.5 M NaOH and protein concentration was determined with the BCA Protein Assay Kit (Pierce). The cells were not scraped off for these experiments.

### *In vivo* study

6 to 8 weeks old female ApoE−/− mice (C57BL/6Jbom-Apoe^tm1Unc^, Taconic M&B laboratory Animals and Services for Research, Ry, Denmark) were fed a western diet (Research Diets, D12079B) for 10, 12, 16, or 17 weeks – time points for which previous studies in our laboratory had shown early lesion formation after 10–12 weeks of diet and advanced lesion formation after 16–17 weeks. Mice were anesthetized and terminally bled followed by perfusion with PBS containing 2% of heparin trough cardiac puncture. Aortas were carefully dissected out and fat tissue removed. For flow cytometry analyses, the whole aorta from the heart down to the kidney branch was used, while the aortic arch from the heart down to the 7^th^ rib were isolated and snap frozen in liquid nitrogen for later RNA extraction.

All animal experiments were performed according to the principles stated in the Danish law on animal experiments and were approved by the Animal Experiment’s Inspectorate, Ministry of Justice, Denmark. The investigation conforms to the Guide for the Care and Use of Laboratory Animals published by the European Parliament [EU directive 2010/63/EU].

### Plasma measurements

Whole blood was sampled from the heart using EDTA as an anticoagulant, centrifuged at 4000 G (rcf) for 8 min. and plasma was kept at −80 °C. Total cholesterol and triglycerides was measured on the Cobas 6000 analyzer (Hoffman La-Roche, Mannheim, Germany).

### RNA extraction and qPCR

RNA from PEMs, BMDMs and atherosclerotic lesions was extracted with RNeasy Lipid Tissue Mini Kit according to the manufacturer’s instructions (cat#74804, Qiagen, Denmark). After 24 hours of oxLDL incubation, the supernatant was discarded, and cells were placed on ice, washed with ice cold PBS, and lysed with Qiazol. The cells were not scraped off for these experiments. Lesion and non-lesion areas were cut out from aortas under a light microscope and homogenized in Qiazol on a Tissuelyzer.

RNA concentration was determined by Nanodrop and cDNA was made using High Capacity cDNA Reverse Transcription Kits (Applied Biosystems). Gene expression was analysed on Custom TaqMan Array Card (Life Technologies) using TaqMan Gene Expression Master Mix (Applied Biosystems) (200 ng RNA/sample corresponding to 4 ng RNA/gene). The primer ID for each of the genes is listed in Suppl. Table 3.

### Flow cytometry analysis

Cells were plated in non-coated plates and stimulated with oxLDL for 24 hours, detached with 4 μM EDTA, very gently scraped off, and resuspended in flow cytometry buffer (PBS without ca/Mg supplemented with 2% FCS and 2 mM EDTA) for further analyses.

Whole aortas were cut into small pieces and incubated at 37 °C for 1 hour under slow shaking in an enzyme cocktail containing 1 mg/mL collagenase I, 1 mg/mL collagenase XI, 60 U/mL hyaluronidase and 60 U/mL DNase I in PBS. The cell suspension was then passed through a 70 μm cell strainer, centrifuged at 400 g for 5 min at 4 °C and resuspended in flow cytometry buffer.

Flow cytometric analysis was performed as follows. Briefly, cells were blocked for unspecific binding with anti-CD16/CD32 (BD), followed by surface staining with F4/80 (BioLegend), CD11c (BD) all diluted in PBS without Ca/Mg supplemented with 2% FCS and 2 mM EDTA. Cells were then fixed and permeabilised using the Cytofix/Cytoperm solution kit (BD) according to the manufacturer’s description and then intracellularly stained for CD68 and CD206 (BD).

Samples were then acquired on a FACS LRSFortessa equipped with blue, red and violet laser followed by data analysis using FACSDiva software (BD). Isotype control antibodies used at a molar access of 3-fold did not give any detectable signal. Gating strategies and dot plots for flow cytometry are shown in Suppl. Figs 3 and 6.

### Statistical analysis

Statistical analyses were performed using GraphPad Prism 6.0 (GraphPad Software Inc., San Diego). Comparisons were done with Student’s *t* test, one-way ANOVA, or two-way ANOVA as specified in the figure legends. Whenever possible (n ≥ 5), data was tested for normality. In cases where data was not normally distributed, a non-parametric test (Mann-Whitney t-test) was used. Statistical analyses applied are specified in legends of figures and tables. *p* < 0.05 was considered significant.

Hotelling’s T^2 tests were performed using the R working environment (www.r-project.org). Arg1 were excluded from the analysis due to a missing data point.

## Additional Information

**How to cite this article**: Bisgaard, L. S. *et al.* Bone marrow-derived and peritoneal macrophages have different inflammatory response to oxLDL and M1/M2 marker expression – implications for atherosclerosis research. *Sci. Rep.* **6**, 35234; doi: 10.1038/srep35234 (2016).

## Supplementary Material

Supplementary Information

## Figures and Tables

**Table 1:** mRNA expression of genes related to M1 and M2 differentiation or lipid metabolism.

Gene of interest	Macrophage (Mø)	Foam cells (Fc)	Foam cell rel. to macrophage	
	PEM rel. to BMDM	PEM rel. to BMDM	BMDM	PEM
M2-associated markers				
Arg1	8757.40**	*58.69*	0.85	**0.01**
Chi3l1	*23.47*	*3.94*	*2.45*	**0.41**
Socs2	42.48****	45.39****	0.74	0.79
CCL17	8691.35****	5058.71****	1.08	0.63
CD206	0.06****	0.09****	1.11	1.64
MGL	0.26*	**0.31**	0.70	0.81
Retnla	N.D.	N.D.	N.D.	N.D.
IL10	N.D.	N.D.	N.D.	N.D.
MGL2	N.D.	N.D.	N.D.	N.D.
IL4	N.D.	N.D.	N.D.	N.D.
CD163	N.D.	N.D.	N.D.	N.D.
M1-associated markers				
IL12a	1384.57**	410.32*	1.08	**0.32**
IL6	381.66*	*46.07*	*2.15*	**0.26**
Ptgs2	46.15***	14.21**	0.58	**0.18**
TNF	4.03**	*2.67*	0.61	0.40*
CCL22	*163.53*	*575.32*	**0.28**	0.98
CD11c	*5.93*	*4.19*	0.79	0.56
Ifnß1	*15.80*	*48.49*	0.62	1.91
Nos2	0.80	*29.82*	0.03	1.14
CXCL10	0.18*	1.60	0.04***	0.33***
Ifnɣ	N.D.	N.D.	N.D.	N.D.
Lipid metabolism				
MSR1	2.40*	1.44	0.71	0.42*
LOX1	2774.26****	980.00****	1.22	**0.43**
SREBP2	2.85**	1.20	0.91	0.38**
ACSL1	*4.01*	1.47	0.95	**0.35**
Abcg1	0.17***	0.56	1.58	5.26***
DGAT2	0.99	0.93	0.32*	**0.31**
SRB1	1.11	0.73	0.61	0.40**
CD36	1.14	1.07	*3.77*	*3.52*
SREBP1	1.57	0.86	2.03*	1.11
Abca1	1.13	0.75	1.94	1.29
DGAT1	1.09	0.91	1.17	0.98
cpt1a	0.91	0.71	1.55	1.20
PPARɣ	0.04***	0.12*	1.11	*3.19*

Column 2 and 3 represent fold induction in PEMs relative to BMDMs grown in either 0 μg/mL oxLDL (macrophage, Mø) or 25 μg/mL oxLDL (foam cells, Fc) for 24 hours. The latter two columns represent the fold induction of gene expression in response to oxLDL stimulation in either BMDMs (column 4) or PEMs (column 5). n = 3 mice for BMDMs and 3 pools of 4–5 mice for PEMs. Two way ANOVA with Tukey’s post-test was applied. *p < 0.5, **p < 0.01, ***p < 0.001, ****p < 0.0001. ND: not detected. Italic >2 fold Bold <0.5 fold blank = no change. Abbreviations: Arginase 1 (Arg1), Chitinase 3-like 1 (Chi3l1), Suppressor of cytokine signaling 2 (Socs2), Chemokine (C-C motif) ligand 17 (CCL17), Mannose receptor, C type 1 (CD206), C-type lectin domain family 10, member A (MGL), Resistin like alpha (Retnla), Interleukin 10 (IL10), Macrophage galactose N-acetyl-galactosamine specific lectin 2 (MGL2), Interleukin 4 (IL4), CD163 antigen (CD163), Interleukin 12a (IL12a), Interleukin 6 (IL6), Prostaglandin-endoperoxide synthase 2 (Ptgs2), Tumor necrosis factor (TNF), Chemokine (C-C motif) ligand 22 (CCL22), Integrin alpha X (CD11c), Interferon beta 1 (Ifnß1), Nitric oxide synthase 2 (Nos2), Chemokine (C-X-C motif) ligand 10 (CXCL10), Interferon gamma (Ifnɣ), Macrophage scavenger receptor 1 (MSR1), Oxidized low density lipoprotein (lectin-like) receptor 1 (LOX1), Sterol Regulatory Element-Binding Protein 2 (SREBP2), Acyl-CoA synthetase long-chain family member 1 (ACSL1), ATP-binding cassette, sub-family G, member 1 (Abcg1), Diacylglycerol O-acyltransferase 2 (DGAT2), Scavenger receptor class B, member 1 (SRB1), CD36 antigen (CD36), Sterol Regulatory Element-Binding Protein 1 (SREBP1), ATP-binding cassette, sub-family A, member 1 (Abca1), Diacylglycerol O-acyltransferase 1 (DGAT1), Carnitine palmitoyltransferase 1a, liver (cpt1a), Peroxisome proliferator-activated receptor gamma (PPARγ).
