# Supplementary material for: Bone marrow-derived and peritoneal macrophages have different inflammatory response to oxLDL and M1/M2 marker expression – implications for atherosclerosis research
Source: Sci Rep. 2016 Oct 13;6:35234. doi: 10.1038/srep35234 (PMC5062347; doi:10.1038/srep35234)
Supplement: Supplementary Information [file srep35234-s1.pdf]

Supplementary

Bone marrow-derived and peritoneal macrophages have different inflammatory response to oxLDL and M1/M2 marker expression – implications for atherosclerosis research

Line S. Bisgaard[1,2], Christina K. Mogensen[2], Alexander Rosendahl[2,4,6], Helena Cucak[2], Lars Bo Nielsen[1,5], Salka E. Rasmussen[3], and Tanja X. Pedersen[1*]

[1]Dept. of Biomedical Sciences, University of Copenhagen, Denmark, [2]Diabetic Complication Biology, [3]ADME, and [4]Biopharmaceuticals New Heamophilia, Novo Nordisk, Denmark, [5]Dept. of Clinical Biochemistry, Copenhagen University Hospital Rigshospitalet, Denmark, [6]Current address Medical Affairs, Shire, Denmark

*Corresponding author

Suppl. table 1. Plasma lipids.

A.	10 week (WD)	17 week (WD)
N	4	4
Triglyceride	2,04 ± 0,08	1,25 ± 0,23*
Cholesterol	31,10 ± 1,86	28,18 ± 1,03
**B.**	**12 week (WD)**	**16 week (WD)**
N	7	7
Triglyceride	1,65 ± 0,14	2,03 ± 0,18
Cholesterol	29,51 ± 1,51	34,48 ± 1,53#

Cholesterol (mM) and triglyceride (mM) levels in ApoE-/- mice used for flow cytometry (A) and gene expression analyses (B) after the indicated time periods on western diet (WD). *$p<0.5$ compared to 10 week, #$p<0.5$ compared to 12 week as determined by students t-test.

Suppl. table 2. mRNA expression of genes related to M1 and M2 differentiation or lipid metabolism in lesion areas isolated from ApoE-/- mice.

	**Lesion area**
Gene of interest	16 week rel. to 12 weeks
**M2 markers**	
Arg1	0.41
Chi3l1	0.19
Socs2	0.61*
CCL17	0.98
CD206	0.52*
MGL	0.56
Retnla	0.23***
IL10	N.D.
MGL2	0.82
IL4	N.D.
CD163	0.54*
**M1-associated markers**	
IL12a	N.D.
IL6	0.44
Ptgs2	0.43
TNF	0.78
CCL22	0.53
CD11c	1.16
Ifnß1	0.99
Nos2	0.57
CXCL10	0.55
Ifnɣ	N.D.
**Lipid metabolism**	
MSR1	0.79
LOX1	1.08
SREBP2	0.63**
ACSL1	0.36**
Abcg1	0.83
DGAT2	0.70
SRB1	0.68*
CD36	0.67
SREBP1	0.85
Abca1	0.87
DGAT1	0.72*
cpt1a	0.59*
PPARɣ	0.80

The column represents fold induction in 16 weeks relative to 12 weeks WD (n=7 mice/group). Student's *t* test was applied, apart from the following genes for which Mann-Whitney tests were applied: MGL, Ptgs2, TNF, MSR1, ACSL1, SREBP1, Abca1 and cpt1a. *p<0.5, **p<0.01, ***p<0.001. ND: not detected.

< 0.1 fold = no change.

Abbreviations: Arginase 1 (Arg1), Chitinase 3-like 1 (Chi3l1), Suppressor of cytokine signaling 2 (Socs2), Chemokine (C-C motif) ligand 17 (CCL17), Mannose receptor, C type 1 (CD206), C-type lectin domain family 10, member A (MGL), Resistin like alpha (Retnla), Interleukin 10 (IL10), Macrophage galactose N-acetyl-galactosamine specific lectin 2 (MGL2), Interleukin 4 (IL4), CD163 antigen (CD163), Interleukin 12a (IL12a), Interleukin 6 (IL6), Prostaglandin-endoperoxide synthase 2 (Ptgs2), Tumor necrosis factor (TNF), Chemokine (C-C motif) ligand 22 (CCL22), Integrin alpha X (CD11c), Interferon beta 1 (Ifnß1), Nitric oxide synthase 2 (Nos2), Chemokine (C-X-C motif) ligand 10 (CXCL10), Interferon gamma (Ifnɣ), Macrophage scavenger receptor 1 (MSR1), Oxidized low density lipoprotein (lectin-like) receptor 1 (LOX1), Sterol Regulatory Element-Binding Protein 2 (SREBP2), Acyl-CoA synthetase long-chain family member 1 (ACSL1), ATP-binding cassette, sub-family G, member 1 (Abcg1), Diacylglycerol O-acyltransferase 2 (DGAT2), Scavenger receptor class B, member 1 (SRB1), CD36 antigen (CD36), Sterol Regulatory Element-Binding Protein 1 (SREBP1), ATP-binding cassette, sub-family A, member 1 (Abca1), Diacylglycerol O-acyltransferase 1 (DGAT1), Carnitine palmitoyltransferase 1a, liver (cpt1a), Peroxisome proliferator-activated receptor gamma (PPARɣ).

Suppl. table 3. Primer ID list

Gene name	Assay ID
Abca1	Mm00442646_m1
Abcg1	Mm00437390_m1
ACSL1	Mm00484217_m1
Arg1	Mm00475988_m1
CCL17	Mm01244826_g1
CCL22	Mm00436439_m1
CD11c	Mm00498698_m1
CD163	Mm00474091_m1
CD206	Mm00485148_m1
CD36	Mm01135198_m1
Chi3l1	Mm00801477_m1
cpt1a	Mm00550438_m1
CXCL10	Mm00445235_m1
DGAT1	Mm00515643_m1
DGAT2	Mm00499536_m1
galectin-3	Mm00802901_m1
Ifnɣ	Mm01168134_m1
Ifnß1	Mm00439552_s1
IL10	Mm00439614_m1
IL12a	Mm00434165_m1
IL4	Mm00445259_m1
IL6	Mm00446190_m1
LOX1	Mm00454586_m1
MGL	Mm00546124_m1
MGL2	Mm00460844_m1
MSR1	Mm00446214_m1
Nos2	Mm00440502_m1
PPARɣ	Mm01184322_m1
Ptgs2	Mm00478374_m1
Retnla	Mm00445109_m1
Socs2	Mm00850544_g1
SRB1	Mm00450234_m1
SREBP1	Mm00550338_m1
SREBP2	Mm01306292_m1
TNF	Mm00443260_g1
18S	Mandatory Company control

Suppl. fig 1. Heat map detailing the magnitude of gene expression

PCR array analyses were used to detect gene expression levels in BMDMs and PEMs grown in either 0 µg/mL oxLDL (macrophages; ctrl) or 25 µg/mL oxLDL (foam cells; oxLDL) for 24 hours (n=3 mice/group for BMDMs, n=3 pools of 4-5 mice for PEMs). Colour bar beneath the heat map indicates the level of expression of each gene ranging from green (minimum expression) over black (average expression) to red (maximum expression).

Suppl. fig 2. Accumulation of macrophages in whole ApoE-/- aortas.

The total number of macrophages (either both $CD68^{+}F4/80^{-}$ and $CD68^{+}F4/80^{+}$ (**A**) or either one of the two subsets (**B**)) (in % of $CD45^{+}$) in the aorta of ApoE-/- mice kept on a western diet (WD) for 10 or 17 weeks (n=4 mice/time point) as determined by flow cytometry. **A**. *p<0.05 as determined by students t-test. **B**. *p<0.05, ****p<0.0001 as determined by 1-way ANOVA with Tukey's post-test.

Suppl. fig 3. Macrophage polarisation in whole ApoE-/- mice

Flow cytometry analysis of whole aortas. Gating strategy (A) and representative mean fluorescence intensity (MFI) histograms for CD206 (B) and CD11c (C). Cells were first identified on SSC vs FSC, singlet cells and living cells determined by 7AAD exclusion. The $CD45^{+}$ leukocytes within this population were then analysed for expression of CD68 and F4/80 and in these sub-populations, expression levels of CD11c and CD206 were determined.

Suppl. fig 4. Galectin-3 expression in aortic lesion vs. non-lesion areas isolated from ApoE-/- mice after 12 or 16 weeks on WD.

mRNA expression of the pan macrophage marker galectin-3 ($2^{-\Delta CT}$) in non-lesion versus lesion areas isolated from ApoE-/- aortas after 12 or 16 weeks on western diet (WD) (n=7 mice/time point). *p<0.05, **p<0.01, as determined by 2-way ANOVA with Tukey's post-test.
Galectin-3: Lectin, galactose binding, soluble 3

Suppl. fig 5. Heat map detailing the magnitude of gene expression *in vivo*

PCR array analyses were used to detect gene expression levels in aortic atherosclerotic lesions isolated from apoE-/- mice after 12 or 16 weeks on western diet (WD) (n=7 mice/group). Colour

bar beneath the heat map indicates the level of expression of each gene ranging from green (minimum expression) over black (average expression) to red (maximum expression).

Suppl. Fig 6. Flow cytometric gating strategy for BMDMs and PEMs

Illustration of the flow cytometric gating strategy of BMDMs (A) and PEMs (B). Singlet cells and live cells were identified as described in supplementary figure 3. $CD45^+$ leukocytes were gated followed by identification of CD68 and F4/80 expression. Based on these markers $CD68^-F4/80^+$ and $CD68^+F4/80^+$ cells were identified and gated upon and their respective CD11c and CD206 expression pattern determined.

Suppl. fig 1

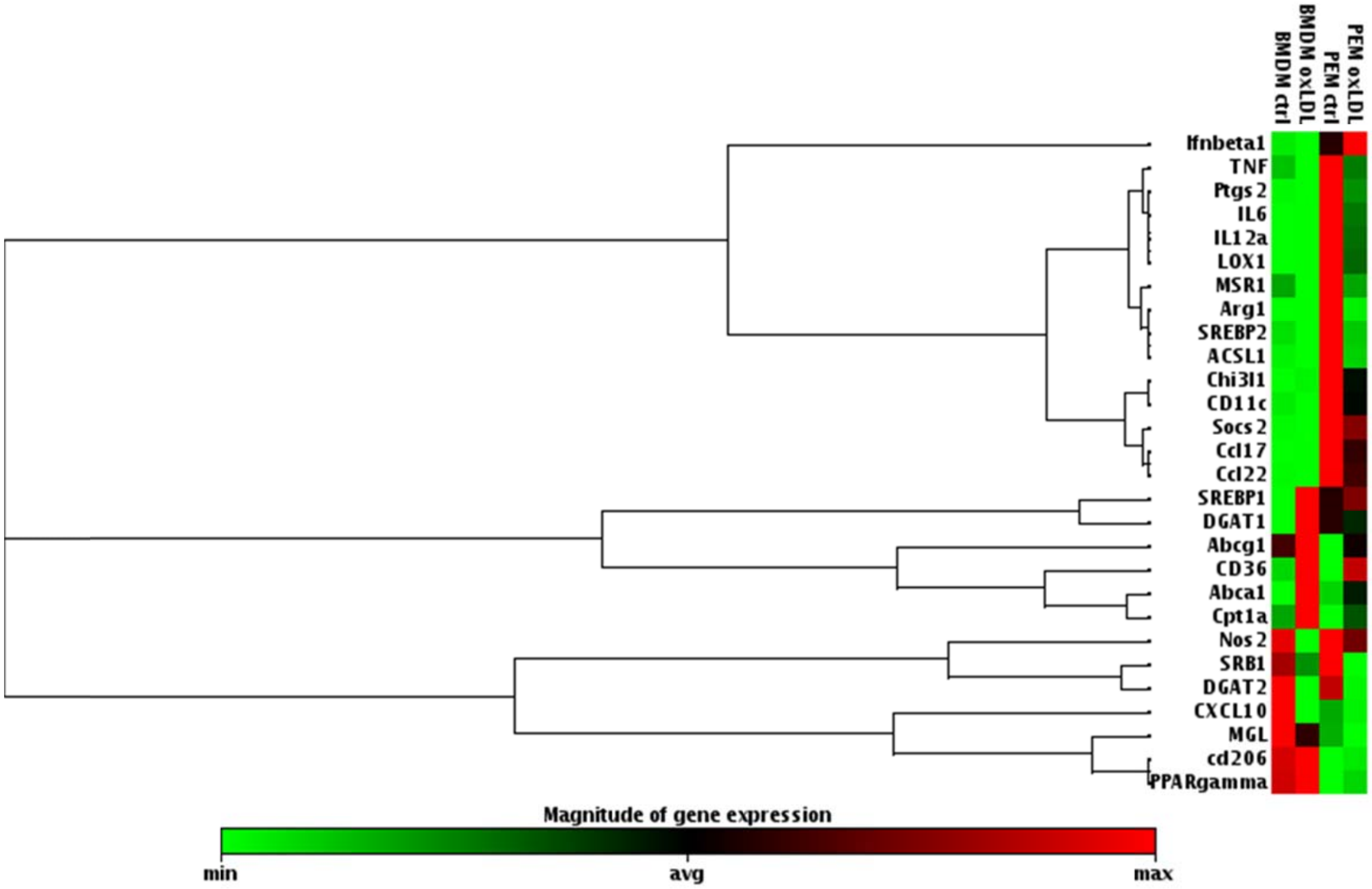

BMDM ctrl
BMDM oxLDL
PEM ctrl
PEM oxLDL
Ifnbeta1
TNF
Ptgs2
IL6
IL12a
LOX1
MSR1
Arg1
SREBP2
ACSL1
Chi3l1
CD11c
Socs2
Ccl17
Ccl22
SREBP1
DGAT1
Abcg1
CD36
Abca1
Cpt1a
Nos2
SRB1
DGAT2
CXCL10
MGL
cd206
PPARgamma
Magnitude of gene expression
min
avg
max

Suppl. fig 2

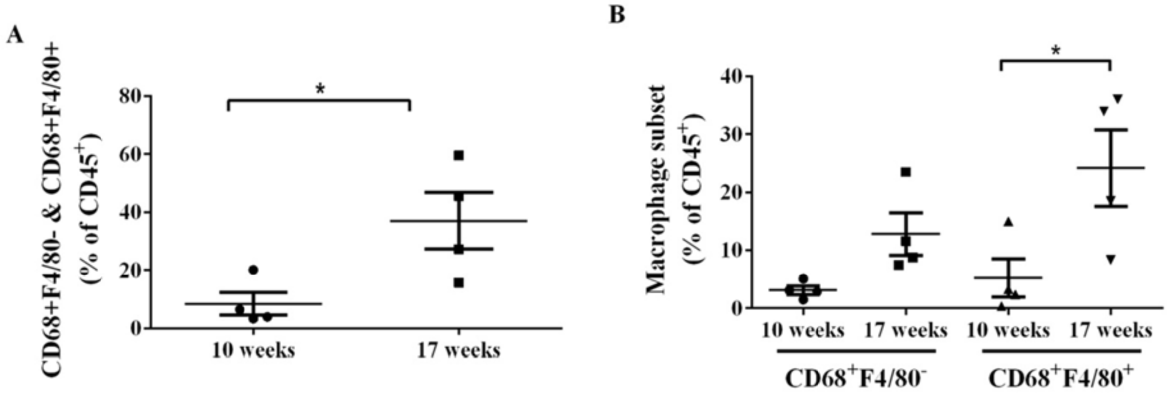

A
CD68+F4/80- & CD68+F4/80+
(% of CD45+)
80
60
40
20
0
*
10 weeks
17 weeks
B
Macrophage subset
(% of CD45+)
40
30
20
10
0
*
10 weeks
17 weeks
10 weeks
17 weeks
CD68+F4/80-
CD68+F4/80+

Suppl. fig 3

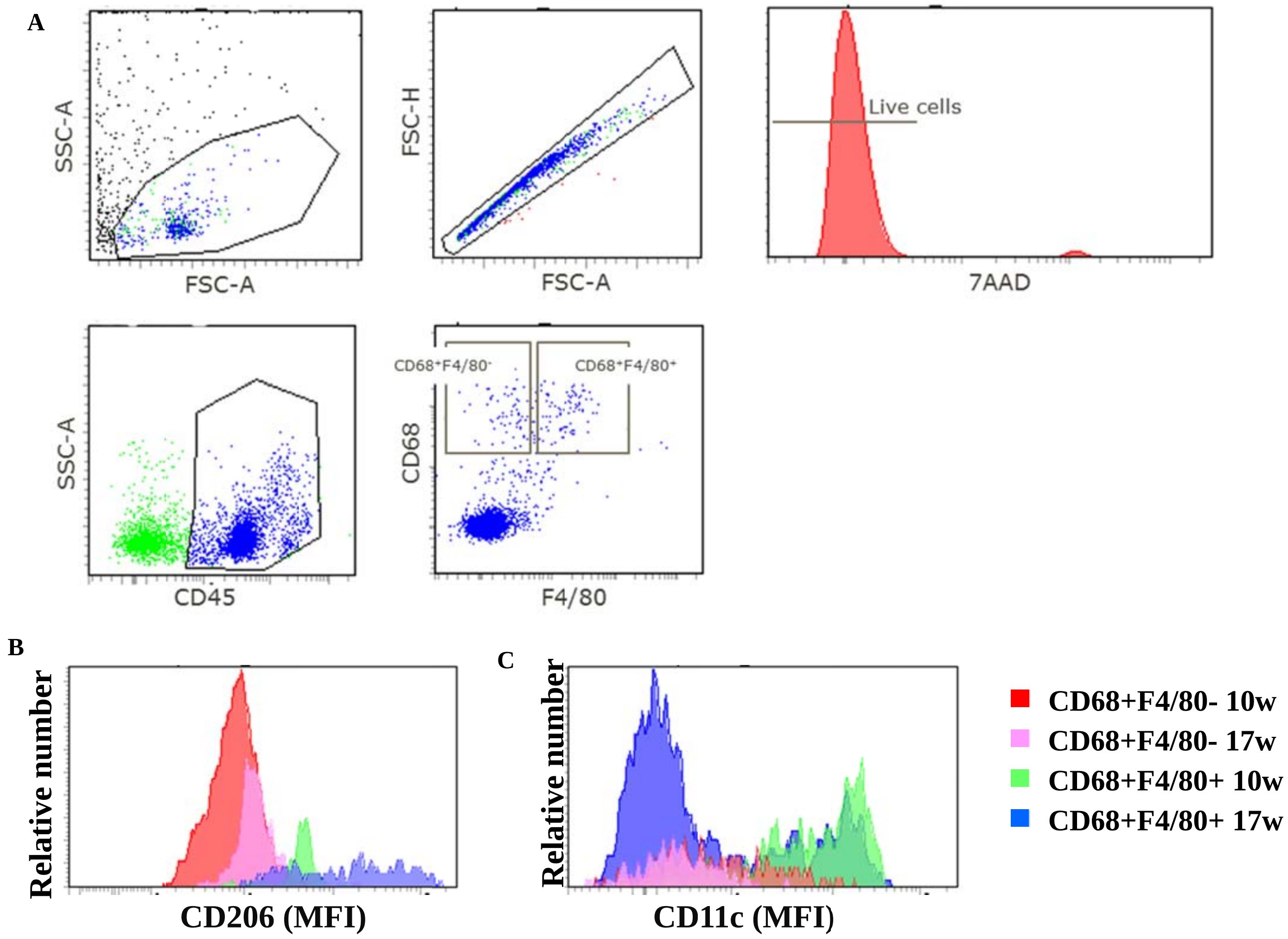
A
SSC-A
FSC-A
FSC-H
FSC-A
Live cells
7AAD
SSC-A
CD45
CD68⁺F4/80⁻
CD68⁺F4/80⁺
CD68
F4/80
B
Relative number
CD206 (MFI)
C
Relative number
CD11c (MFI)
CD68+F4/80- 10w
CD68+F4/80- 17w
CD68+F4/80+ 10w
CD68+F4/80+ 17w

Suppl. fig 4

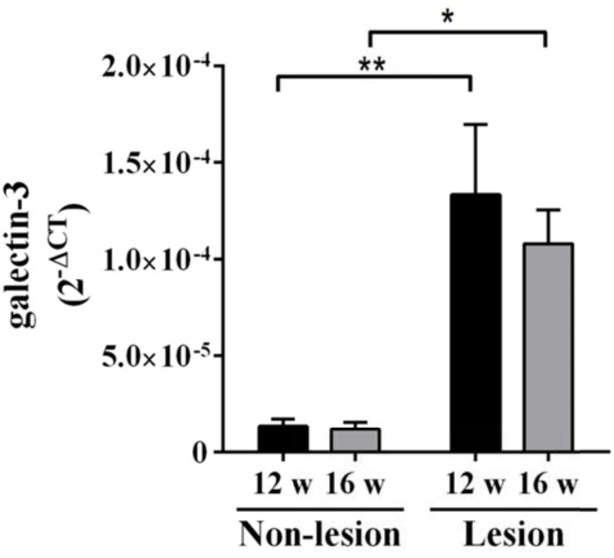

*
**
2.0×10⁻⁴
1.5×10⁻⁴
1.0×10⁻⁴
5.0×10⁻⁵
0
galectin-3
(2^-ΔCT)
12 w 16 w
12 w 16 w
Non-lesion
Lesion

Suppl. fig 5

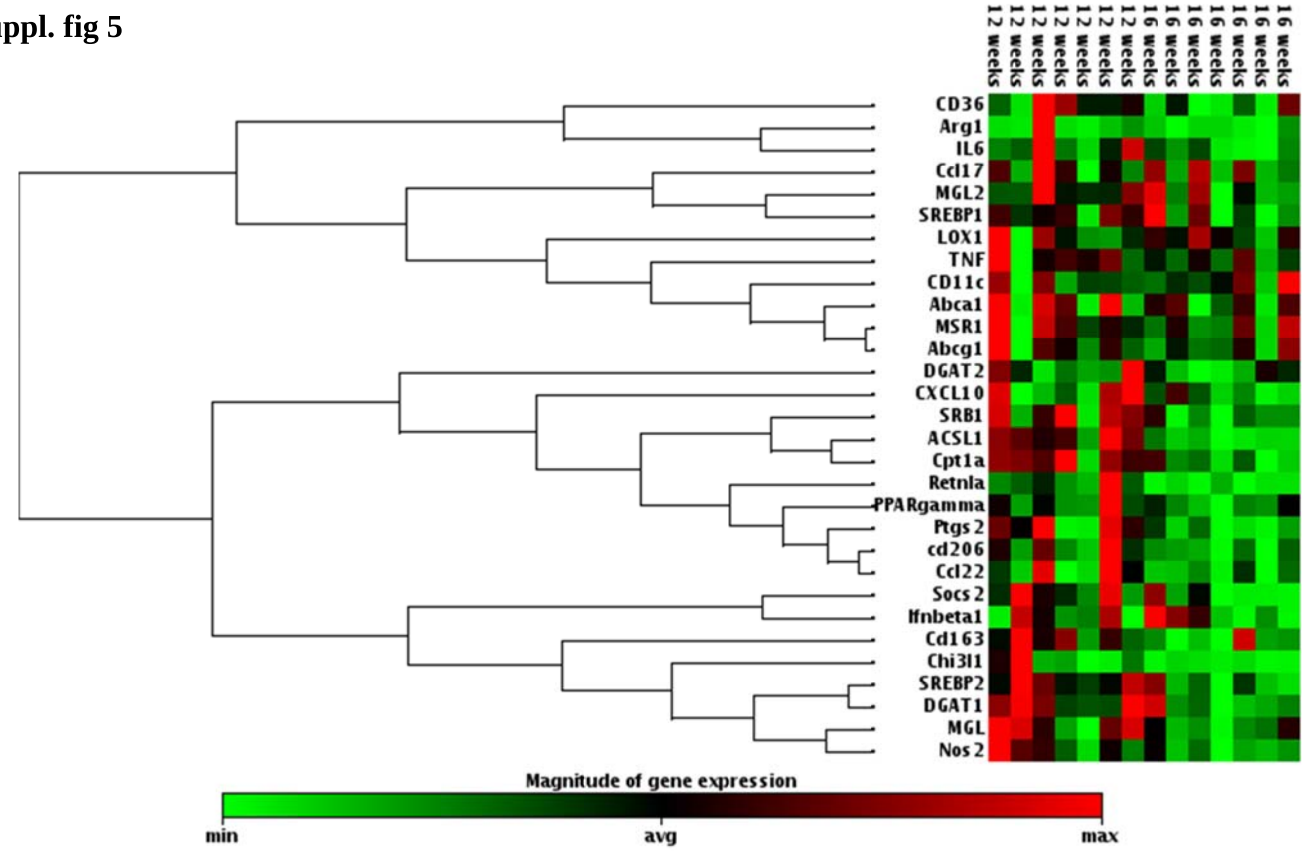

12 weeks
12 weeks
12 weeks
12 weeks
12 weeks
12 weeks
12 weeks
16 weeks
16 weeks
16 weeks
16 weeks
16 weeks
16 weeks
16 weeks
CD36
Arg1
IL6
Ccl17
MGL2
SREBP1
LOX1
TNF
CD11c
Abca1
MSR1
Abcg1
DGAT2
CXCL10
SRB1
ACSL1
Cpt1a
Retnla
PPARgamma
Ptgs2
cd206
Ccl22
Socs2
Ifnbeta1
Cd163
Chi3l1
SREBP2
DGAT1
MGL
Nos2
Magnitude of gene expression
min
avg
max

Suppl. fig 6

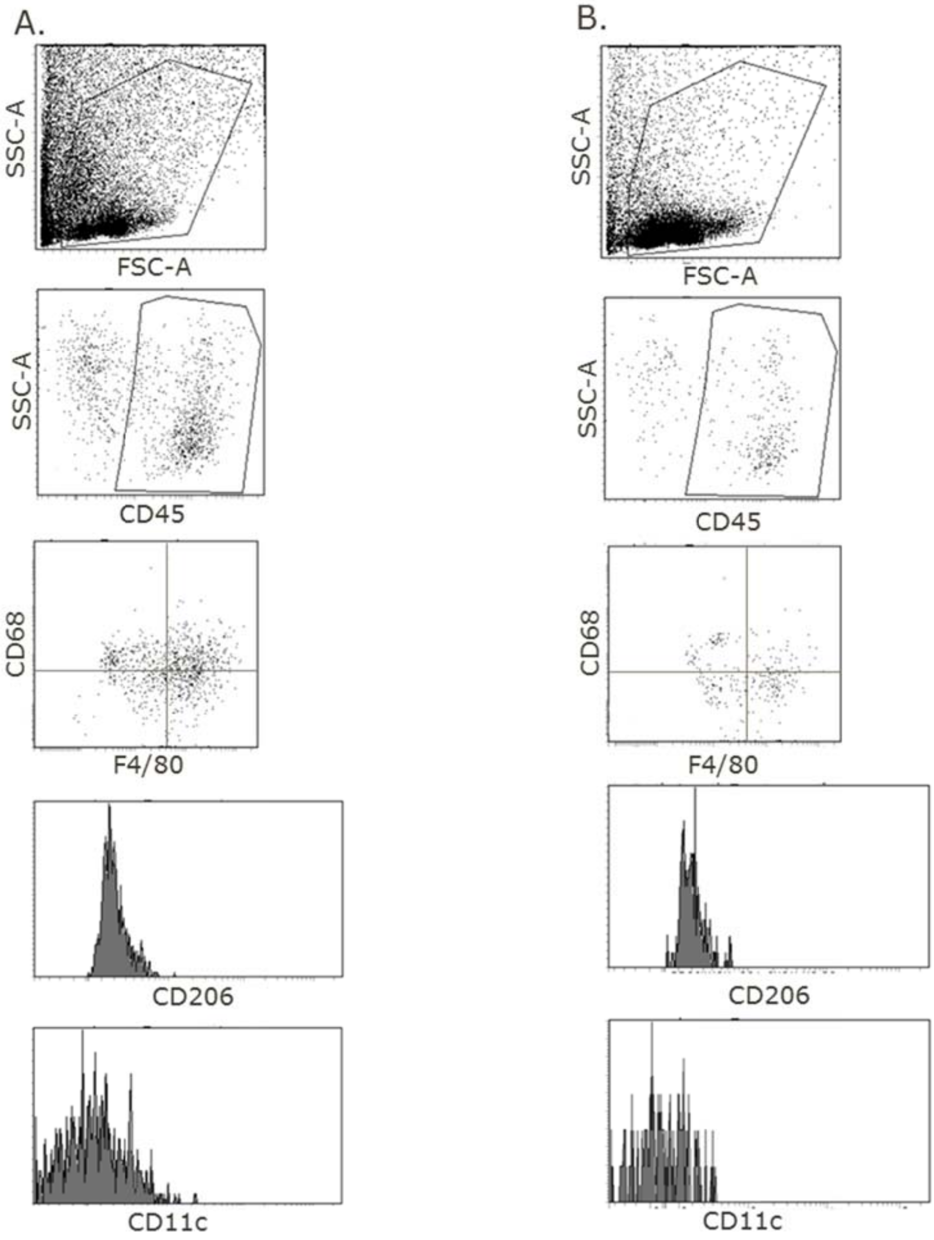

A.
SSC-A
FSC-A
SSC-A
CD45
CD68
F4/80
CD206
CD11c
B.
SSC-A
FSC-A
SSC-A
CD45
CD68
F4/80
CD206
CD11c
